# A Case of Cytomegalovirus Colitis in an Immunocompetent COVID-19 Patient

**DOI:** 10.7759/cureus.23203

**Published:** 2022-03-16

**Authors:** Mohamad Bakir, Fatima Rebh, Elham Mahdi, Abdullah AlFiaar, Gaafar S Mansoor.

**Affiliations:** 1 College of Medicine, Alfaisal University, Riyadh, SAU; 2 Department of Internal Medicine, Section of Infectious Diseases, Prince Mohammed Bin Abdulaziz Hospital, Riyadh, SAU; 3 Department of Pathology, Prince Mohammed Bin Abdulaziz Hospital, Riyadh, SAU; 4 Department of Gastroenterology and Hepatology, Prince Mohammed Bin Abdulaziz Hospital, Riyadh, SAU

**Keywords:** case report, immunocompetent, infection, cmv colitis, cytomegalovirus (cmv), sars‐cov‐2, coronavirus, covid-19

## Abstract

Cytomegalovirus (CMV) is a double-stranded DNA virus that belongs to the herpesvirus family. In the immunocompetent host, CMV infection is usually mild and goes unnoticed. Patients become prone to CMV infection as a result of immunosuppressive drugs or disorders that weaken cellular immunity. In severe COVID-19 infection, the patient experiences a drop in his T lymphocytes and becomes prone to opportunistic infections such as CMV colitis. In this paper, we presented a rare case of CMV colitis in a 54-year-old female with a positive severe acute respiratory syndrome coronavirus 2 (SARS-CoV2) polymerase chain reaction. The patient was admitted to the intensive care unit and intubated due to the severity of her presentation. The patient received high-dose dexamethasone followed by a tapering dose of prednisolone. Fifteen days post-admission, the patient started to have melena with a drop in her hemoglobin. Sigmoidoscopy revealed ulcerated lesions that extended 5 cm proximally, and multiple biopsies confirmed the diagnosis of CMV colitis. The patient was started on ganciclovir 5 mg/kg intravenously for 21 days. The patient's symptoms improved to the point where she no longer complained of melena, and her hemoglobin level normalized. The patient was discharged home in stable condition, to be followed later in the outpatient clinic.

## Introduction

The Severe Acute Respiratory Syndrome Coronavirus 2 (SARS‐CoV‐2), a novel coronavirus, causes Corona Virus Disease-19 (COVID-19). The coronavirus pandemic has been designated as a public health emergency of international concern by the World Health Organization (WHO) [1]. Respiratory viruses, including Middle East respiratory syndrome coronavirus (MERS-CoV), seasonal influenza, and SARS-CoV-1, exhibit varying degrees of bacterial and fungal co-infection [2]. As a result, there is a clinical need for thorough research on co-infection in COVID-19 patients. Cytomegaloviruses (CMV), which are members of the *Herpesviridae* family, are extensively disseminated; nonetheless, these viruses are rarely responsible for major illness or impairment in healthy, nonpregnant adults [3]. CMV infections pose a major risk to patients with disseminated cancer, allograft recipients, or those who are immunocompromised [3]. According to a meta-analysis, 7% of hospitalized COVID-19 patients had a bacterial co-infection, which increased to 14% in studies that only included Intensive care unit (ICU) patients [2]. In addition, the meta-analysis estimated that 3% of COVID-19 patients were additionally infected with another respiratory virus, with influenza A and respiratory syncytial virus (RSV) being the most common [2]. However, there are only a few reported cases in the literature talking about cytomegalovirus colitis co-infection in COVID-19 patients. According to recent research, SARS-CoV-2 infection may largely disrupt T lymphocytes, specifically CD4+ and CD8+ T cells, which may be heavily involved in the pathological process of COVID-19 [4]. In this paper, we present a rare case of CMV colitis infection in a COVID-19 patient who is otherwise immunocompetent.

## Case presentation

**Figure 1:**
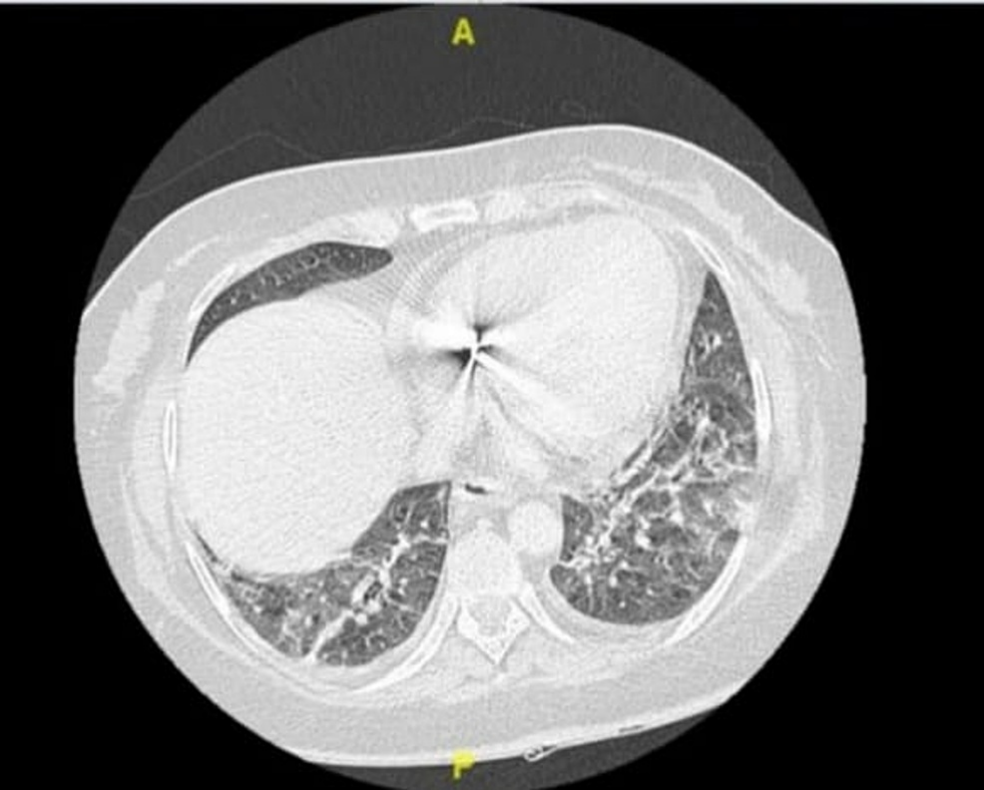
Computed tomography (CT) scan showing bilateral lower lung lobes patchy ground-glass opacification and atelectasis.

**Figure 2:**
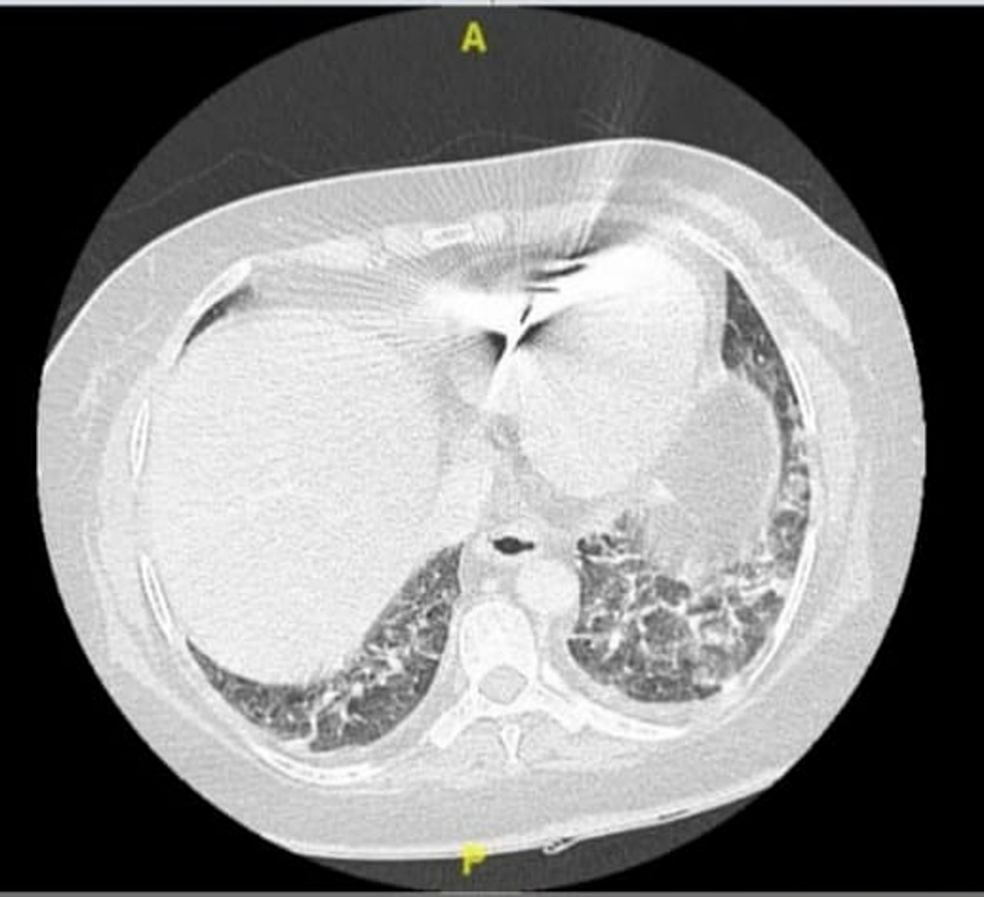
Computed tomography (CT) scan showing bilateral lower lung lobes patchy ground-glass opacification and atelectasis.

A 54-year-old female patient known to have diabetes mellitus, hypertension, and ischemic heart disease presented with respiratory symptoms and a positive SARS-CoV2 polymerase chain reaction (PCR) test. Upon presentation, the patient was hypotensive and had a partial pressure of oxygen (PO2) of 88%. Accordingly, she was admitted to the intensive care unit (ICU), and intubated for 6 days. She received high-dose dexamethasone 4 mg/kg intravenously for 10 days and then switched to a tapering dose of prednisolone over 21 days. The patient did not receive tocilizumab or remsidevir. After 10 days of admission to the ICU, the patient improved and was transferred to the regular ward. Fifteen days post-admission, the primary nurse noticed melena during changing the patient's diaper. In the following days, she kept passing a small amount of black stool with a drop in hemoglobin from 12 g/L to 8 g/L. The patient denied any history of hematemesis, abdominal pain, or distension. Whole-body computed tomography (CT) displayed bilateral lower lung lobes patchy ground-glass opacification and atelectasis (Figures 1, 2).

**Figure 3:**
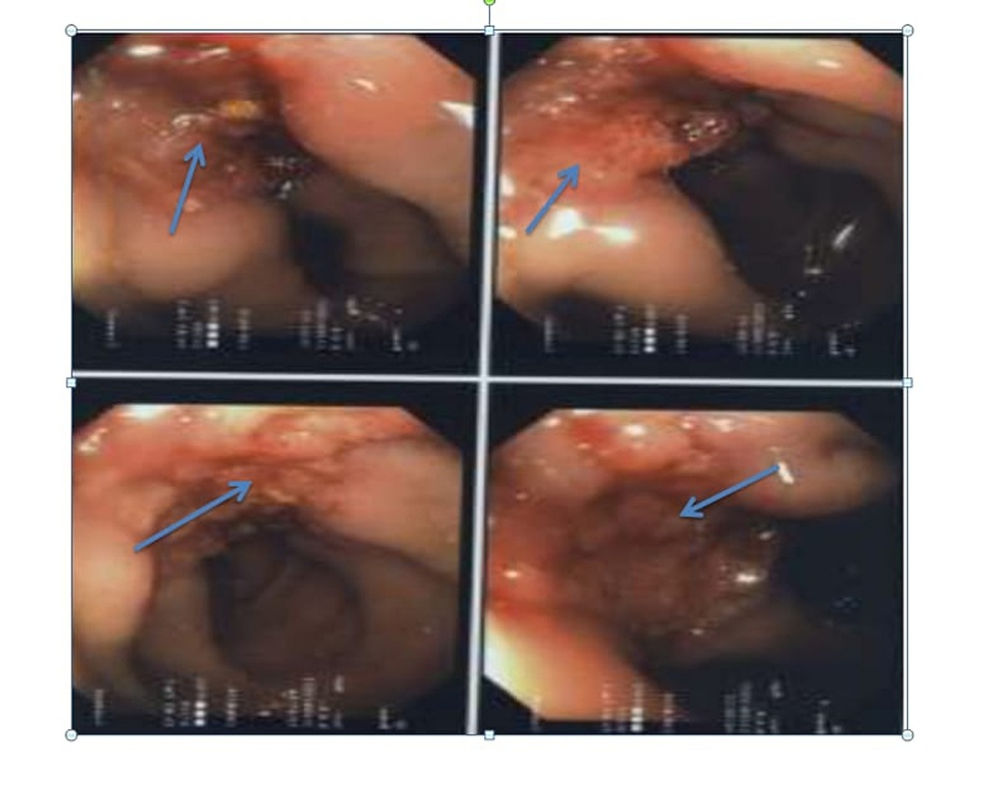
The sigmoidoscopy is showing multiple irregular, map-like (geographical) erosions and ulcers with multiple polypoidal-like lesions (arrows).

Abdominal and pelvic organs were normal apart from multiple bilateral cortical cysts in both kidneys. The gastroenterology team was consulted, and they proceeded with sigmoidoscopy, which revealed ulcerated lesions that extend 5 cm proximally from the sigmoid colon (Figure 3), and due to poor preparation, the total colonoscopy was not completed.

**Figure 4:**
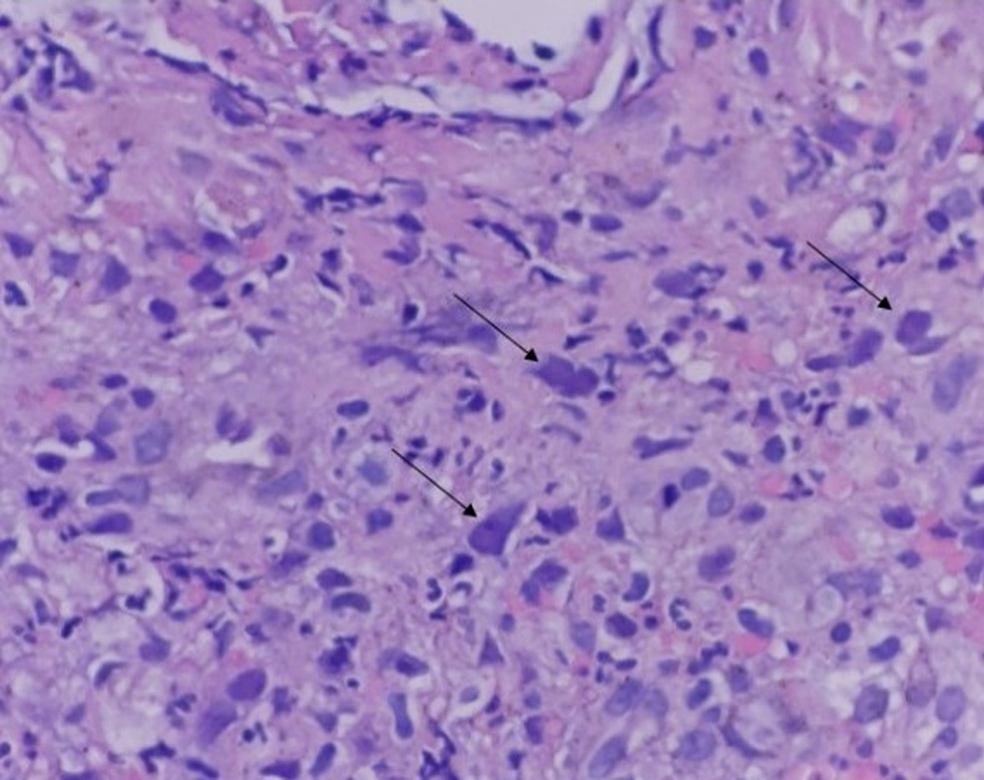
Hematoxylin and Eosin (H&E) stain showing CMV cytopathic changes (arrows). Magnification 400x. CMV: cytomegalovirus

**Figure 5:**
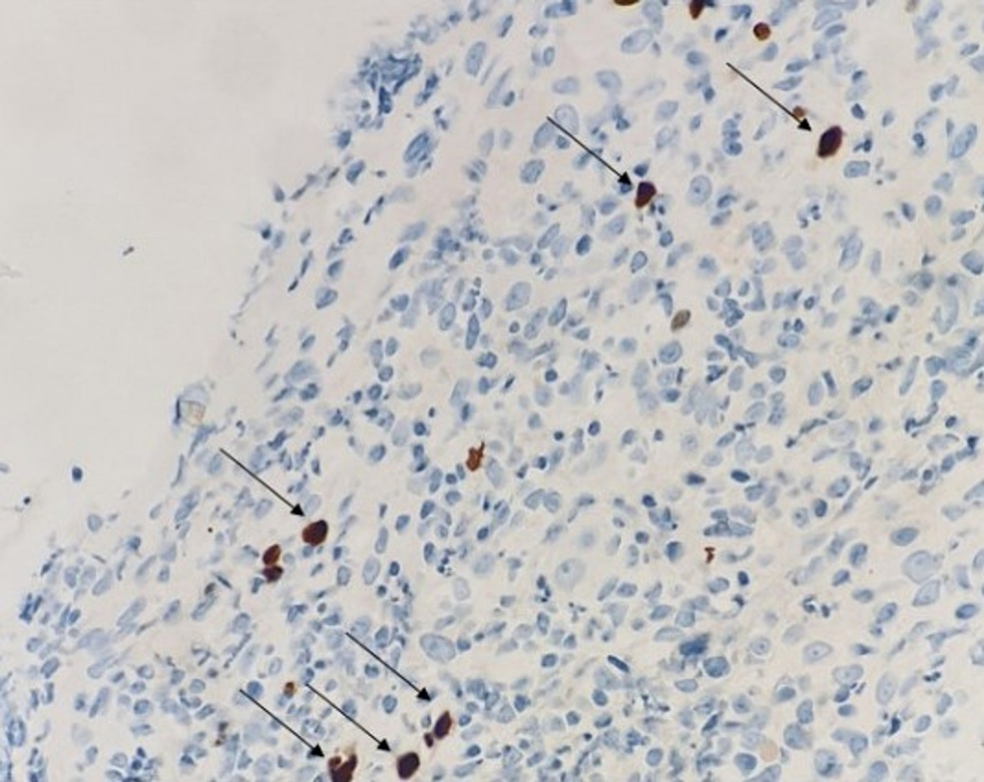
Immunohistochemistry stain (IHC) for cytomegalovirus antibody (CMV AB) (DAKO Clone CCFH2+ DDG9 Ready for Autostainer) - brown color (arrows). Magnification 400x.

Multiple biopsies were taken from the ulcerated lesions, and they were sent for histopathology, which showed dense granulation tissue with chronic and acute inflammation with few stromal cells showing suspicious viral-like CMV cytopathic changes in form of nuclear inclusions (Figure 4). The cytopathic features were confirmed to be CMV using a CMV-specific antibody (CMV AB) immunohistochemistry stain (DAKO Clone CCFH2+ DDG9 Ready for Autostainer [Agilent, Santa Clara, USA]) in our pathology laboratory with pertinent positive and negative controls (Figure 5). 

The CMV antigenemia assay was negative, and carcinoembryonic antigen (CEA) was within the normal range. Other tests, such as human immunodeficiency virus (HIV) testing and blood culture, came back negative. The patient was diagnosed with CMV colitis, and she was started on ganciclovir 5 mg/kg intravenously for 21 days. The patient's symptoms improved to the point where she no longer complained of melena, and her hemoglobin level improved and stabilized at 11 g/L. The patient was discharged home in stable condition, to be followed later in the outpatient clinic. 

## Discussion

CMV is a double-stranded DNA virus that belongs to the *Herpesviridae* family. The CMV genome (about 230 kb) is the largest among human viruses, with 200 genes encoding proteins [5]. CMV colitis is normally asymptomatic or produces self-limiting disease in healthy people, but it can lead to chronic infection or a life-long carrier state with intermittent reactivation. Cytomegalovirus colitis is more common in immunocompromised patients, such as those with AIDS, hematological malignancy, organ transplantation, corticosteroid therapy, and cancer therapy [5]. In immunocompetent patients, CMV colitis should be included in the differential diagnosis, especially the elderly with comorbidities who come with hematochezia, in particular, if they are getting red blood cell transfusions or receiving corticosteroids [5]. CMV colitis is characterized by nonspecific symptoms such as diarrhea, fever, abdominal pain, weight loss, and rectal bleeding. The most common symptoms in these patients are hematochezia and diarrhea [5]. The prevalence of CMV in the general population, as shown by serology, is 70% in adults and reaches 100% in poor and developing countries [6]. CMV colitis is diagnosed through histological evaluation of biopsy tissues collected from the ulcer's margin or base. Colonic mucosal biopsies would show the characteristic inclusions associated with CMV colitis on hematoxylin and eosin (H & E) stain. However, Since H & E staining has low sensitivity [5], the diagnosis has to be confirmed by immunohistochemistry, which is considered the gold standard for diagnosing CMV colitis. Because of the severity of antiviral drug side effects, the majority of patients with CMV colitis who are immunocompetent may not require antiviral treatment, and there is no proof that antiviral treatment will make a significant difference in patient outcomes [5]. Ganciclovir, either orally or intravenously, is the drug of choice in CMV colitis [7]. Although CMV colitis has a great overall prognosis, various risk factors for poor prognosis and higher mortality have been identified, such as old age, male gender, patients requiring surgery, and patients with CMV colitis reactivation in association with ulcerative colitis [5]. Complications of CMV colitis include chronic inflammation, toxic megacolon, ischemic colitis, large bowel perforation, pseudo-membrane formation, and severe hemorrhage [5,8,9]. In COVID-19, It is believed that the use of corticosteroids, prolonged mechanical ventilation, and lymphopenia are the main risk factors for CMV reactivation [10,11]. In this paper, we presented a case of CMV colitis in a middle-aged female who was immunocompetent and had just recovered from a COVID-19 infection. It is important to put CMV colitis in the differential diagnosis of COVID-19 patients who present with hematochezia and diarrhea since SARS-CoV-2 decreases the numbers of CD4+ and CD8+ T lymphocytes, especially in severe cases. In addition, corticosteroids, which are an important part of the treatment protocol for COVID-19 infection, suppress immunity and increase the risk of opportunistic infections.

## Conclusions

Coinfections coexist in many cases, complicating the clinical picture in COVID-19. Coinfections are particularly present in severe cases, which are accompanied by low lymphocyte counts and a higher risk of opportunistic infections, which are rarely encountered in immunocompetent patients. Even though cytomegaloviruses (CMV) are widely distributed, these viruses are rarely responsible for severe illness or impairment in healthy individuals. However, this is not the case for severe COVID-19 infection, where the patient is more prone to opportunistic infections secondary to the disease process or the treatment of COVID-19 infection such as corticosteroids and immunomodulators. Therefore, early identification of risk factors for severe COVID-19 infection is critical because it allows for proper supportive care and prompt treatment since the complications of CMV colitis can be deadly. 
